# The association of triglyceride levels with the incidence of initial and recurrent acute pancreatitis

**DOI:** 10.1186/s12944-021-01488-8

**Published:** 2021-07-18

**Authors:** Robert J. Sanchez, Wenzhen Ge, Wenhui Wei, Manish P. Ponda, Robert S. Rosenson

**Affiliations:** 1 Regeneron Pharmaceuticals, Inc., Tarrytown, New York, USA; 2 Mount Sinai Heart, Icahn School of Medicine at Mount Sinai, One Gustav L. Levy Place, Hospital Box 1030, New York, NY 10029 USA

**Keywords:** Acute pancreatitis, Hypertriglyceridemia, Recurrent acute pancreatitis, Susceptibility threshold, Triglycerides

## Abstract

**Background:**

This retrospective cohort study assessed the annualized incidence rate (IR) of acute pancreatitis (AP) in a nationally representative US adult population, as well as the variation in the risk of AP events across strata of triglyceride (TG) levels.

**Methods:**

Data were obtained from IQVIA’s US Ambulatory Electronic Medical Records (EMR) database linked with its LRxDx Open Claims database. Inclusion criteria included ≥1 serum TG value during the overlapping study period of the EMR and claims databases, ≥1 claim in the 12-month baseline period, and ≥ 1 claim in the 12 months post index. All TG measurements were assigned to the highest category reached: < 2.26, ≥2.26 to ≤5.65, > 5.65 to ≤9.94, > 9.94, and > 11.29 mmol/L (< 200, ≥200 to ≤500, > 500 to ≤880, > 880, and > 1000 mg/dL, respectively). The outcome of interest was AP, defined as a hospitalization event with AP as the principal diagnosis.

**Results:**

In total, 7,119,195 patients met the inclusion/exclusion criteria, of whom 4158 (0.058%) had ≥1 AP events in the prior 12 months. Most patients (83%) had TGs < 2.26 mmol/L (< 200 mg/dL), while < 1% had TGs > 9.94 mmol/L (> 880 mg/dL). Overall, the IR of AP was low (0.08%; 95% confidence internal [CI], 0.08–0.08%), but increased with increasing TGs (0.08% in TGs < 2.26 mmol/L [< 200 mg/dL] to 1.21% in TGs > 11.29 mmol/L [> 1000 mg/dL]). In patients with a prior history of AP, the IR of AP increased dramatically; patients with ≥2 AP events at baseline had an IR of 29.98% (95% CI, 25.1–34.9%).

**Conclusion:**

The risk of AP increases with increasing TG strata; however, the risk increases dramatically among patients with a recent history of AP.

## Background

Annual incidence rates (IRs) of acute pancreatitis (AP) are increasing worldwide [1]. In the United States, the incidence of AP is approximately 110 to 140 per 100,000 person-years, resulting in more than 300,000 emergency visits yearly [2, 3] with medical expenditures of $7000 per hospitalized episode [4, 5]. Many patients with AP remain at high risk for recurrent episodes, and may develop chronic pancreatitis [4, 5]. Long-term consequences of chronic pancreatitis include exocrine insufficiency in 38–40% of cases, and diabetes in 38–40% of cases [1, 6–8]. In a meta-analysis of 14 studies including 8492 patients with AP, the pooled prevalence of AP and chronic pancreatitis was 22 and 10%, respectively [5].

Hypertriglyceridemia is considered causal in up to 10% of AP episodes [9]. These estimates were derived from convenient cohorts of patients. However, despite the fact that hypertriglyceridemia is an established risk factor for AP, few nationally representative data are available on IRs of AP across varying strata of TG levels. Few studies have evaluated the effects of TG on the outcome of AP [10]; however, TGs measured at the time of hospitalization for AP may be lower than the concentration that triggered the acute event due to prolonged fasting following the onset of abdominal pain, particularly in patients with a prior episode of AP. Further, it remains uncertain whether IRs for AP differ by TG level among patients with an initial vs. recurrent hospitalization for AP. Most studies that have examined the association between TG levels and AP have been conducted in retrospective analyses, with the exception of one prospective, population-based study of patients with moderate hypetriglyceridemia that was conducted in Denmark [11]. The purpose of this study was to examine the IR of AP across strata of TG levels and by history of AP using large administrative healthcare databases in the US.

## Methods

This study used de-identified patient data in accordance with established privacy guidelines under the Health Insurance Portability and Accountability Act. Therefore, the study was exempt under the Department of Health and Human Services policy 45 CFR 46.101(b)(4) and a separate institutional review board approval or patient informed consent were not sought.

This retrospective cohort study utilized data from IQVIA’s US Ambulatory Electronic Medical Records (EMR) database linked with its LRxDx Open Claims database between January 2006 and May 2020. IQVIA US Ambulatory EMR database contains anonymized patient medical records from outpatient visits covering ~ 76 million patients in the US. The LRxDx open claims database contains anonymized patient records collected by physicians during an office visit to document patients’ clinical records and their pre-adjudicated inpatient/outpatient claims submitted by physicians/specialists for the purpose of reimbursement. Inclusion criteria included ≥1 serum TG value during the overlapping study period of the EMR and claims databases, ≥1 claim during the 12-month baseline period, and ≥ 1 claim in the 12 months post index. Patients with a history of alcoholism (International Classification of Diseases [ICD], Ninth Edition [ICD9] code 303.x, ICD, 10th Edition [ICD10] codes F10.1x, F10.2x) or gallstone diseases (ICD9 code 574x, ICD10 code K80x) were excluded. All TG measurements (fasting status unknown) for each patient were identified from their EMR and assigned to the highest category reached: < 2.26, ≥2.26 to ≤5.65, > 5.65 to ≤9.94, > 9.94, and > 11.29 mmol/L (< 200, ≥200 to ≤500, > 500 to ≤880, > 880, and > 1000 mg/dL, respectively). These TG thresholds were selected as they are associated with an increased risk of AP by various consensus statements (≥5.65 mmol/L [≥500 mg/dL], American Heart Association/American College of Cardiology cholesterol guidelines [12]; ≥9.94 mmol/L [≥880 mg/dL], European Society of Cardiology/European Atherosclerosis Society guidelines [13]; ≥11.29 mmol/L [≥1000 mg/dL], American Association of Clinical Endocrinologists/American College of Endocrinology consensus statement [14]). If patients had more than one TG value from the highest TG stratum, a random measurement was selected as the index TG (exposure variable); this was defined as the date of the index TG. The outcome of interest was the first AP event, defined as a hospitalization event with AP as the principal diagnosis (ICD9 code 577.0 or ICD10 code K85x). Baseline history of AP was based on the total number of AP events (using the same definition as the outcome; only AP events that are at least 7 days apart were counted) during the baseline period and patients were grouped into having 0, 1, and ≥ 2 AP events. Patients were followed up for 1 year. Baseline age, sex, comorbididties, and history of AP were described by TG group. IRs and 95% confidence intervals (CIs) of AP were calculated both overall and by TG group, as well as by baseline history of AP. IRs of AP were calculated as the number of AP events divided by the total number of person-years of follow-up.

## Results

**Fig. 1:**
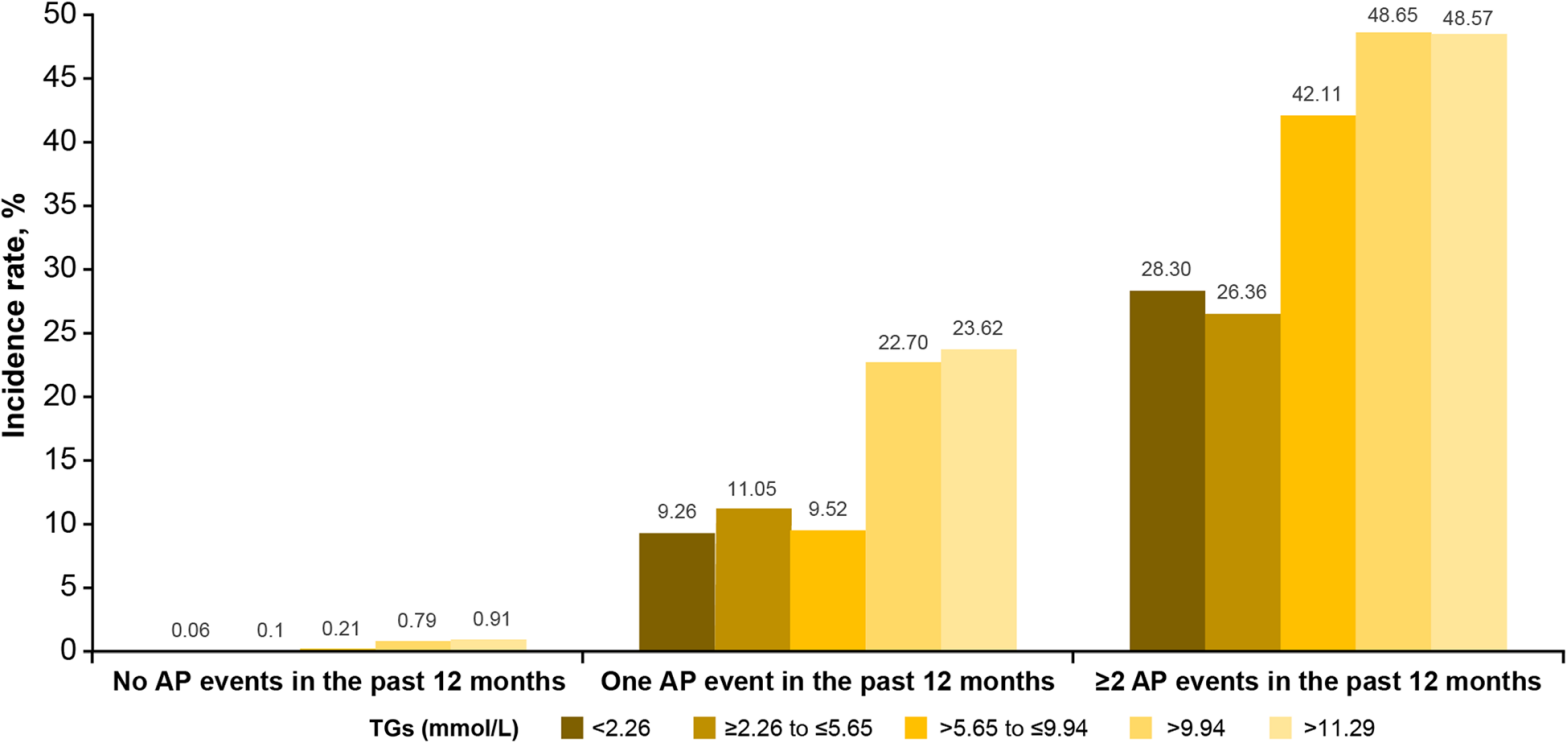
Overview of IR of AP by circulating TG concentration and history of hospitalization for AP. *Abbreviations: AP* acute pancreatitis, *CI* confidence interval, *IR* incidence rate, *TG* triglyceride

There were 7,119,195 patients who met the inclusion/exclusion criteria, of whom 4158 (0.058%) had one or more AP events in the prior 12 months. The average age of the cohort was 54.2 (± 18.2 years) and 45% were male (Table 1). The majority of patients (83%) had TGs < 2.26 mmol/L (< 200 mg/dL), while < 1% had TG values > 9.94 mmol/L (> 880 mg/dL). In the overall population, the annualized IR of AP was low at 0.08% (95% CI, 0.08–0.08%); however, the IR increased with increasing TGs, ranging from 0.08% in TGs < 2.26 mmol/L (< 200 mg/dL) to 1.21% in TGs > 11.29 mmol/L (> 1000 mg/dL) (Table 2 and Fig. 1). When examining the IR by history of AP events, the IR was low among those with no prior history (0.07, 95% CI 0.07–0.07%). However, the IR increased dramatically in those with a history of AP, with those with ≥2 events at baseline having an IR of 29.98% (95% CI, 25.1–34.9%). Patients with ≥2 AP events at baseline and TGs > 11.29 mmol/L (> 1000 mg/dL) had the highest IR, 48.57% (95% CI, 31.72–65.72%).

**Table 1:** Baseline characteristics

	TGs mmol/L (mg/dL)					
	All	< 2.26 (< 200)	≥2.26–≤5.65 (≥200–≤500)	> 5.65–≤9.94 (> 500–≤880)	> 9.94(> 880)	> 11.29(> 1000)
Patients, *n*	7,119,195	5,914,102	1,111,737	72,665	20,691	15,281
Age, years, mean (SD)	54.23 (18.2)	53.89 (18.02)	56.68 (14.98)	53.82 (13.17)	51.28 (11.78)	50.78 (11.32)
Male, n (%)	3,213,139 (45)	2,582,688 (44)	567,319 (51)	48,032 (66)	15,100 (73)	10,869 (71)
TGs, mg/dL, mean (SD)	112.8 (299.3)	67.2 (57.9)	277.8 (61.2)	678 (95.8)	1429.4 (3122.4)	1672.6 (3907.2)
Baseline acute pancreatitis events, n (%)						
0	7,115,037 (99.94)	5,911,134 (99.95)	1,110,849 (99.92)	72,541 (99.83)	20,513 (99.14)	15,119 (98.94)
1	3681 (0.05)	2657 (0.04)	778 (0.07)	105 (0.14)	141 (0.68)	127 (0.83)
≥ 2	477 (0.01)	311 (0.01)	110 (0.01)	19 (0.03)	37 (0.18)	35 (0.23)
Baseline diabetes mellitus, n (%)	1,006,571 (14.14)	712,726 (12.05)	263,230 (23.68)	24,656 (33.93)	5959 (28.80)	1816 (11.88)
Baseline steatosis of liver, n (%)	63,763 (0.90)	41,656 (0.70)	19,240 (1.73)	2130 (2.93)	737 (3.56)	132 (0.86)
Baseline obesity, n (%)	3,044,051 (42.76)	2,545,273 (43.04)	454,548 (40.89)	32,005 (44.04)	12,225 (59.08)	9622 (62.97)

*Abbreviations: SD* standard deviation, *TG* triglyceride

**Table 2:** IR of AP by circulating TG concentration and history of hospitalization for AP

	AP			
	Total, ***N***	Cases, ***n***	Crude IR, % (95% CI)	Relative risk (95% CI)
All patients				
Overall	7,119,195	5686	0.08 (0.08–0.08)	
TGs < 2.26 mmol/L (< 200 mg/dL)	5,914,102	4111	0.07 (0.07–0.07)	Reference
TGs ≥2.26–≤5.64 mmol/L (≥200–≤500 mg/dL)	1,111,737	1194	0.11 (0.10–0.11)	1.55 (1.45–1.65)
TGs > 5.65–≤9.94 mmol/L (> 500–≤880 mg/dL)	72,665	169	0.23 (0.20–0.27)	3.35 (2.87–3.90)
TGs > 9.94 mmol/L (> 880 mg/dL)	20,691	212	1.02 (0.89–1.17)	14.74 (12.84–16.92)
TGs > 11.29 mmol/L (> 1000 mg/dL)	15,281	185	1.21 (1.05–1.40)	17.42 (15.03–20.18)
No AP event in the past 12 months				
Overall	7,115,037	5169	0.07 (0.07–0.07)	
TGs < 2.26 mmol/L (< 200 mg/dL)	5,911,134	3777	0.06 (0.06–0.07)	Reference
TGs ≥2.26–≤5.65 mmol/L (≥200–≤500 mg/dL)	1,110,849	1079	0.10 (0.09–0.10)	1.52 (1.42–1.63)
TGs > 5.65–≤9.94 mmol/L (> 500–≤880 mg/dL)	72,541	151	0.21 (0.18–0.24)	3.26 (2.77–3.83)
TGs > 9.94 mmol/L (> 880 mg/dL)	20,513	162	0.79 (0.68–0.92)	12.36 (10.56–14.46)
TGs > 11.29 mmol/L (> 1000 mg/dL)	15,119	138	0.91 (0.77–1.08)	14.28 (12.05–16.93)
One AP event in the past 12 months				
Overall	3681	374	10.16 (9.1–11.2)	
TGs < 2.26 mmol/L (< 200 mg/dL)	2657	246	9.26 (8.20–10.44)	Reference
TGs ≥2.26–≤5.65 mmol/L (≥200–≤500 mg/dL)	778	86	11.05 (8.98–13.52)	1.19 (0.93–1.53)
TGs > 5.65–≤9.94 mmol/L (> 500–≤880 mg/dL)	105	10	9.52 (4.91–17.22)	1.03 (0.55–1.94)
TGs > 9.94 mmol/L (> 880 mg/dL)	141	32	22.70 (16.25–30.66)	2.45 (1.70–3.54)
TGs > 11.29 mmol/L (> 1000 mg/dL)	127	30	23.62 (16.74–32.14)	2.55 (1.75–3.73)
≥2 AP events in the past 12 months				
Overall	477	143	29.98 (25.1–34.9)	
TGs < 2.26 mmol/L (< 200 mg/dL)	311	88	28.30 (23.43–33.71)	Reference
TGs ≥2.26–≤5.65 mmol/L (≥200–≤500 mg/dL)	110	29	26.36 (18.63–35.78)	0.93 (0.61–1.42)
TGs > 5.65–≤9.94 mmol/L (> 500–≤880 mg/dL)	19	8	42.11 (21.12–66.03)	1.49 (0.72–3.07)
TGs > 9.94 mmol/L (> 880 mg/dL)	37	18	48.65 (32.24–65.33)	1.72 (1.04–2.85)
TGs > 11.29 mmol/L (> 1000 mg/dL)	35	17	48.57 (31.72–65.72)	1.72 (1.02–2.89)

*Abbreviations: AP* acute pancreatitis, *CI* confidence interval, *IR* incidence rate, *TG* triglyceride

## Discussion

IRs for AP increased with higher TG levels; however, the IR of AP among patients with hypertriglyceridemia and no prior episodes of AP was low, in striking contrast to reports from multiple small cohort studies summarized in consensus statements on hypertriglyceridemia [9]. Overall, the distribution of TGs in the current study population was similar to a prevalence study of TGs in US adults, supporting the generalizability of these findings [15]. Novel aspects of the current study were the association between TGs obtained before hospitalization for AP and evaluation of TGs associated with AP before initial and recurrent hospitalization for AP. Among patients with TGs > 11.29 mmol/L (> 1000 mg/dL), IRs for AP were < 1%, but increased markedly with each prior episode of AP, and at lower TG levels. A prospective study of 116,500 Danes reported an IR for AP of 0.12% with TGs between 5.00–9.99 mmol/L (443–885 mg/dL) [11], which is lower compared to the result from the current study. Several factors might have contributed to this discordance, including outcome identification and differences of other unmeasured risk factors for AP, sample size, and increasing incidence of AP in the past decades.

Variability in the IR of AP in patients with TGs < 880 mg/dL can be explained by variability in post-prandial clearance rates of TG-rich lipoproteins (TGRLs). TG levels > 880 mg/dL are associated with persistent chylomicronemia, whereas chylomicrons are not consistently observed at lower TG levels. Chylomicrons or large TGRL remnants may contribute to the risk of AP because of their size, independent of their TG content per se. In this case, patients with TGs < 880 mg/dL would be expected to have greater variability in concentration and size of chylomicrons or chylomicron remnants, especially in the post-prandial state. This variability, then, would translate into variability into AP event rates in this strata of TG levels.

The increased risk of AP among patients with a past history of AP at lower TGs requires further research. However, several possibilities for this finding include increased susceptibility from prior pancreatic injury, as well as self-management by patients with prior hospitalizations for AP who may attempt to manage their symptoms and delay hospitalization by prolonged fasting and use of pain medications. The association between lower TG levels and AP may result from very low-density lipoprotein-mediated elevations in blood viscosity [16] that impede perfusion in the microcirculation of the pancreas [17].

### Strengths and limitations

Strengths of the study include large numbers of patients from a nationally representative population of US adults, the exclusion of patients with gallstones and alcoholism, and a large volume of data on recurrent episodes of AP. Gallstones are the most common cause of AP, accounting for 40–70% of cases [18], while alcohol consumption is responsible for 25–35% of cases [19]. Limitations included the nature of the database, which prohibited comprehensive adjustment of confounders; exposure miclassification being possible as a single TG measurement was relied upon; etiology of AP being unknown; and misclassification of AP being possible due to coding errors. These limitations include an inability to distinguish AP from an acute attack of chronic pancreatitis, though clinically this may be a distinction without a difference in terms of risk of recurrence of acute and severe pancreatic disease. Further, alcohol consumption is likely to be under-reported and under-coded in databases; therefore, it is possible that the underlying cause of AP associated with hypertriglyceridemia is under-captured.

The influence of TG-lowering therapies on the TGs at the time of the hospitalization was unable to be determined. However, it is recognized that many patients with severe abdominal pain may not consume TG-lowering and other medications. Extant therapeutics are unlikely to achieve the substantial (> 50%) TG lowering required to mitigate the impact of hypertriglyceridemia on the risk of recurrent pancreatitis, underscoring the need to study novel therapeutics and the potential benefit conferred by lowering TG levels.

## Conclusions

These data show that the IR of AP increases with increasing TG levels, and importantly, in patients with a prior history of AP, a dramatic increase in the IR of a future AP episode. In addition, these data highlight the importance of considering lower TG susceptibility thresholds in the evaluation of patients with recurrent vs. initial AP episodes, and provide guidance on TG levels for clinical studies designed to prevent TG-mediated acute and chronic pancreatitis.

## Data Availability

Not applicable.

## Abbreviations

AP: Acute pancreatitis
CI: Confidence interval
EMR: Electronic medical record
ICD: International Classification of Diseases
IR: Incidence rate
TG: Triglyceride
